# Functional Limitations in Stroke Survivors: Pre-Stroke Physical Activity Matters

**DOI:** 10.1101/2023.09.14.23295576

**Published:** 2023-09-18

**Authors:** Zack Van Allen, Dan Orsholits, Matthieu P. Boisgontier

**Affiliations:** 1 School of Rehabilitation Sciences, Faculty of Health Sciences, University of Ottawa, Canada; 2 Perley Health Centre of Excellence in Frailty-Informed Care, Ottawa, Canada; 3 Bruyère Research Institute, Ottawa, Canada

**Keywords:** Cohort studies, Comorbidity, Disability, Exercise, Functional status, Health behavior, Longitudinal studies, Prognosis, Prospective studies, Stroke survivors

## Abstract

**Background and Purpose.:**

In the chronic phase after a stroke, limitations in activities of daily living (ADL) and instrumental ADL (IADL) initially plateau before steadily increasing. However, the benefits of pre-stroke levels of physical activity on these limitations remain unclear. To clarify this relationship, this study compares the effect of physical activity on the long-term evolution of I/ADL limitations between stroke survivors and stroke-free controls.

**Methods.:**

Longitudinal data from 2,143 stroke survivors and 10,717 matched stroke-free controls aged 50 and over were drawn from the Survey of Health, Ageing and Retirement in Europe (SHARE; 2004–2020). Physical activity was assessed on the wave preceding the stroke event and number of I/ADL limitations during the post-stroke chronic phase. Each stroke survivor was matched with 5 stroke-free controls who had similar propensity scores that were computed based on key covariates. The effect of pre-stroke physical activity on I/ADL limitations in stroke survivors was compared to its effect in stroke-free controls with a similar time lag between physical activity and I/ADL assessments using linear mixed-effects models adjusted for age, sex, education level, and the number of chronic conditions.

**Results.:**

In stroke survivors, the beneficial effect of pre-stroke physical activity on ADL limitations after stroke is significantly stronger than its effect in stroke-free controls matched for baseline age, sex, body mass index, limitations in I/ADL, chronic conditions, and country of residence, before any of the participants had experienced a stroke.

**Conclusions.:**

Physical activity is an effective preventive intervention that reduces the risk of functional dependence after stroke. In addition, pre-stroke level of physical activity is an important variable in the prognosis of functional dependence after stroke.

Each year, the prevalence of stroke exceeds 100 million cases worldwide. On average, each of these cases is associated with a loss of 1.4 year of full health^1,2^. Over the past three decades, the number of years of full health lost to stroke has increased by an average of 1.2 million per year1. This burden on stroke survivors is reflected in their functional limitations. Specifically, one year after a stroke, 59%^3–17^, 33%^13–28^, and 23%^11–13,15–20^ of survivors experience at least slight, moderate, or severe dependency in activities of daily living (ADLs), respectively, such as dressing, walking, bathing, eating, and toileting (Table 1–3). Regarding instrumental ADLs (IADLs), 40%^9,10,16,19,20^ of stroke survivors are moderately active and 41%^16,17,19–21^ are inactive in domestic chores, leisure, work, and outdoor activities at one year (Table 4–5). Whether limitations in I/ADLs plateau^10,13,21,28,29^ or increase^11,12,19^ in subsequent years depends on several factors, including age^11,12,29,30^, type of health insurance^11^, and severity of disability 1 to 2 years after stroke^12^.

The level of physical activity has been suggested as one of the factors influencing functional limitations after stroke^31^. Regarding ADLs, some studies have found an association between higher prestroke physical activity and lower post-stroke disability in ADLs^22,32–37^. Specifically, higher pre-stroke physical activity was associated with higher independence in ADLs during the first^22, 32–36^ and second year^37^ after stroke. However, other studies found no evidence of this association between physical activity and functional independence in ADLs^38–41^. These mixed results could be explained by the use of a single-item rating scale^22,32,33,35–41^, the Modified Rankin Scale, which has been shown to be less reliable and more subjective than questionnaires assessing specific I/ADLs^42^. In addition, only one prospective study has examined the effect of physical activity before stroke on IADLs^30^. This study focused on vigorous physical activity and was based on a cohort of adults who were stroke-free at baseline. The results showed that higher vigorous physical activity at baseline was associated with a higher probability of being independent in I/ADLs after stroke, but this difference was similar before stroke. This result led the authors to conclude that “being physically active does not protect against the disabling effects of a stroke” on I/ADLs. Building on this previous study, we used a different approach by comparing the effect of physical activity on I/ADLs in a larger sample of stroke survivors (n = 2,143 vs. 1,374) with a sample of stroke-free controls matched for key covariates (n = 10,717). Moreover, because it has been suggested that moderate-intensity physical activity is at least as beneficial to brain plasticity as vigorous-intensity physical activity^43,44^, we included both intensities.

In this prospective cohort study, we hypothesized that the beneficial effect of pre-stroke moderate-to-vigorous physical activity on I/ADL limitations after stroke would be significantly stronger than its effect in stroke-free controls matched for baseline (i.e., before any of the participants had experienced a stroke) age, sex, body mass index, I/ADL limitations, and country of residence over a similar number of follow-up years.

## Methods

### Study Sample and Design

Data were drawn from the Survey of Health, Ageing and Retirement in Europe (SHARE), a longitudinal population-based study on adults 50 years of age or older living in 28 European countries and one Middle East country^45^. Data were collected every two years between 2004 and 2020 for a total of 8 measurement waves using computer-assisted personal interviewing (CAPI) in participants’ homes. Physical activity, stroke events, and functional independence (ADLs, IADLs) were assessed at all measurement waves except wave 3 (2008–2009). To be included in the present study, participants had to be 50 years of age or older, have never reported having a stroke before entering the study, and have participated in at least 4 waves. SHARE was carried out in accordance with the Declaration of Helsinki and has been approved by the Ethics Committee of the University of Mannheim (waves 1–4) and the Ethics Council of the Max Plank Society (waves 4–8). All participants provided written informed consent.

### Measures

#### Outcome variable: Functional limitations

Functional dependence was assessed using the number of functional dependencies in six ADLs (dressing, walking, bathing, eating, getting in or out of bed, and using the toilet) and seven IADLs (using a map, preparing a hot meal, shopping for groceries, making telephone calls, taking medication, gardening or doing housework, and managing money)^46,47^. Higher scores were indicative of higher functional dependence.

#### Explanatory variables: Stroke and physical activity

Information on stroke status during follow-up was collected at each wave using the following question: “Has a doctor told you that you have any of the conditions on this card [indicating history of health conditions including stroke]?”^12^.

The level of physical activity at entry in SHARE was derived from two questions: “How often do you engage in vigorous physical activity, such as sports, heavy housework, or a job that involves physical labor?” and “How often do you engage in activities that require a low or moderate level of energy such as gardening, cleaning the car, or doing a walk?”^47–52^. Participants answered using a four-point scale: 1 = Hardly ever or never; 2 = One to three times a month; 3 = Once a week; 4 = More than once a week. Participants who answered “more than once a week” to at least one of the questions were classified as physically active, whereas the other participants were classified as physically inactive to reduce a potential misclassification bias in which physically inactive participants would be wrongly classified as physically active.

#### Covariates

Models were adjusted for baseline age, sex (male, female), time (survey waves), quadratic time, number of chronic conditions (none or 1 vs. 2 or more), and level of education, which has shown to be associated with the level of physical activity^48,51,53–57^.

### Data Preprocessing

#### Matching procedure

To select matched samples of stroke survivors and stroke-free participants with similar distributions of key covariates, a matching procedure based on the nearest neighbor method was conducted using the MatchIt R package^58,59^ with propensity scores obtained with a generalized linear model. This matching process used a 1:5 ratio to create groups including one stroke survivor and five stroke-free controls with similar propensity scores, thereby reducing the potential bias introduced by covariates. Propensity scores were calculated using characteristics of the participants at their first interview for the Survey of Health, Ageing and Retirement in Europe (SHARE), i.e., when none of them had experienced a stroke: Age, sex, number of chronic conditions (none or 1 vs. 2 or more), limitations in I/ADL, body mass index category [underweight (below 18.5 kg/m^2^), normal (reference; 18.5 to 24.9 kg/m^2^), overweight (25 to 29.9 kg/m^2^), obese (30 kg/m^2^ and above)], country of residence, number of measurement waves, and wave number of the first interview.

#### Statistical analyses

Data were analyzed using linear mixed-effects models that account for the nested structure of the data (i.e., repeated measurement over time within a single participant) and provide acceptable Type I error rates^60^. The models were built and fit by maximum likelihood in R programming language^61^ using the lme4^62^ and lmerTest^63^ packages. P-values were approximated using the Satterthwaite’s method^64^. Specifically, to investigate the effect of pre-stroke physical activity on functional independence in stroke survivors and stroke-free controls, two dependent variables were tested: ADL and IADL limitations. The fitted models included stroke (stroke vs. no stroke), physical activity (active vs. inactive at baseline), linear time, quadratic time, and the covariates as fixed effects. The random structure encompassed random intercepts for participants and for participants grouped together by the matching process as well as random linear and quadratic slopes for the repeated measurements at the level of participants. These random effects estimated each participant’s and each matching group’s functional independence as well as the rate of change of this independence over time. The quadratic effect of age was added to account for the potential accelerated (or decelerated) decline of functional independence across time. An interaction terms between stroke and physical activity was added to formally test the moderating effect of stroke on the association between physical activity and functional dependence.

#### Sensitivity analysis

In a sensitivity analysis, participants who answered “hardly ever or never” to one of the two questions related to the level of physical activity were classified as physically inactive, whereas the other participants were classified as physically active. This categorization reduced a potential misclassification bias in which physically active participants would wrongly be classified as physically inactive.

## Results

The study sample included 2,143 stroke survivors (mean age: 66.9 ± 9.1 years; 1,052 females) and 10,717 stroke-free controls (mean age: 66.9 ± 9.3 years, 5,126 females) whose characteristics at baseline are summarized in Table 6.

**Figure 1.**
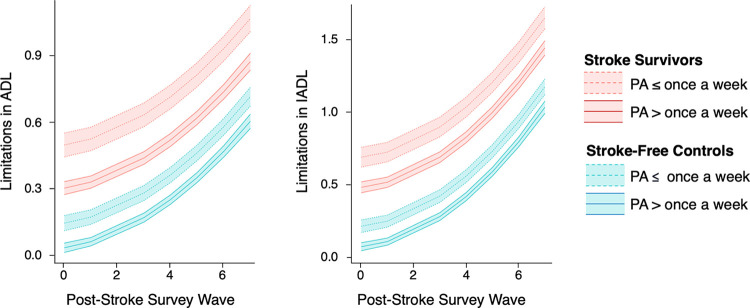
Effect of physical activity (PA: once a week or less vs. more than once a week) on limitations in activities of daily living (ADL) and instrumental activities of daily living (IADL) in stroke survivors and matched stroke-free controls over time.

Results of the mixed-effects models showed an interaction effect between stroke and physical activity on limitations in ADL (b = 0.083, 95% confidence interval [CI]: 0.018 to 0.148, p = 0.013; Table 7, Figure 1). The simple effects of the terms of this interaction confirmed that the effect of physical activity was stronger in stroke survivors (b = 0.268, 95% CI: 0.241 to 0.296, p < 2.0 × 10^−16^) than in stroke-free controls (b = 0.351, 95% CI: 0.292 to 0.411, p < 2.0 × 10^−16^).

For IADL, results showed no evidence of an interaction effect between stroke and physical activity on limitations in IADL (b = 0.067, 95% CI: −0.016 to 0.149, p = 0.149; Table 7, Figure 1).

**Figure 2.**
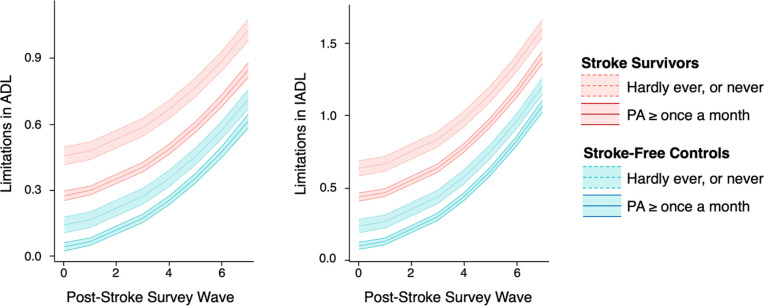
Result of the sensitivity analysis showing the effect of physical activity (PA: Hardly ever or never vs. at least once a month) on limitations in activities of daily living (ADL) and instrumental activities of daily living (IADL) in stroke survivors and matched stroke-free controls over time.

Results of sensitivity analyses were consistent with the results to the main analyses (Table 8; Figure 2).

## Discussion

### Main Results

The results of this large cross-national longitudinal study suggest that the beneficial effect of pre-stroke physical activity on in ADL limitations after stroke is significantly stronger than its effect in stroke-free controls matched for age, sex, body mass index, limitations in I/ADLs, chronic conditions, and country of residence, before any of the participants had experienced a stroke.

### Comparison With Other Studies

Our results showed that higher levels of pre-stroke physical activity were associated with fewer ADL limitations. These fin dings are in line with the existing literature showing that an association between higher pre-stroke physical activity and lower post-stroke disability in ADLs^22,32–37^. Our findings support these results. Most importantly, they reveal that the effect of pre-stroke physical activity on in ADL limitations after stroke is significantly stronger than its effect in matched stroke-free controls. While the study by Ris et al.^30^ also examined the effect of physical activity on both stroke survivors and stroke-free controls (without the matching procedure we conducted), this potential interaction effect was not considered.

Several mechanisms could explain how physical activity enhances post-stroke functional independence. This effect could be explained by an association between pre- and post-stroke physical activity as previous studies showed that this level was similar in 41 to 42% of stroke survivors activity^65,66^. This post-stroke engagement in physical activity could increase brain plasticity processes such as angiogenesis, synaptogenesis, and neurogenesis, primarily through the upregulation of growth factors (e.g., brain-derived neuro- trophic factor; BDNF)^67–69^. However, the same studies also showed that 33 to 39% of stroke survivors reported lower physical activity after compared to before stroke, and 20 to 25% reported higher physical activity^65,66^. Another explanation could be the protective effect of pre-stroke physical activity on depression^65^, which has shown to be associated with ADL limitations^47,70,71^.

### Strengths and Limitations

The present study has several strengths including results presented for a long follow-up period (up to 16 years) and a large international post-stroke population (17 countries), which allowed us to robustly examine the effects of physical activity on I/ADL limitations. The number of I/ADL limitations used to evaluate changes in functional limitation over time, which are more reliable than single-item rating and more sensitive to identify differences in functional trajectory between stroke cases and controls. The sensitivity results using different categories for physical activity were consistent with the main results.

However, our findings should be considered in light of several limitations. (1) There was a lack of information on stroke subtypes, which is common in and inherent to large-scale longitudinal studies. Future studies should be supported by medical records to provide a more specific understanding of the relationship between physical activity and functional independence in stroke survivors. (2) The outcome (i.e., stroke) was self-reported. Therefore, a memory bias cannot be excluded. However, the agreement between self-reported stroke and medical records ranges from 79%^72^ to 96%^73^. (3) Physical activity was self-reported, which may not have accurately captured the actual levels of physical activity, as correlations between self-report and direct measures of physical activity are low to moderate^74,75^. Future studies should assess physical activity using device-based measures, as they have shown greater validity and reliability^76^.

## Conclusion

Our findings support a stronger long-term beneficial effect of physical activity on independence in ADLs in stroke survivors compared with stroke-free adults. These findings underscore the essential preventive role of moderate-to-vigorous physical activity in mitigating stroke-related limitations in ADLs. In addition, these findings highlight the need to consider the pre-stroke levels of physical activity in the prognosis of stroke-related functional independence.

## Figures and Tables

**Table 1.** Stroke survivors with at least slight dependency in activities of daily living (ADLs) at 1 year follow-up.

Study	Outcome Measure	Threshold	Sample Size (n)	Dependent Survivors (%)

Appelros (2007)	Barthel Index	<20/20	246	39.0
Ayerbe (2011)	Barthel Index	<20/20	1732	67.0
Carolei (1997)	Barthel Index	<20/20	517	61.7
Dhamoon (2009)	Barthel Index	<95/100	525	48.1
Gil-Salcedo (2022)	Modified Ranking Scale	>1/6	3718	63.8
Hartman-Maeir (2007)	FIM motor scale	<91/91	56	68.0
Leśniak (2008)	Barthel Index	<20/20	80	43.7
Mar (2015)	Barthel Index	<100/100	250	47.2
Minelli (2007)	Barthel Index	<100/100	79	57.0
Skånér (2007)	Katz ADL	<6/6	135	31.9
Sveen (1996)	Barthel Index	<20/20	74	58.1
Taub (1994)	Barthel Index	<20/20	225	34.0
van de Port (2006)	Barthel Index	<19/20	264	40.1
Willey (2010)	Barthel Index	<95/100	246	44.7
Wong (2014)	Modified Ranking Scale	>1/6	194	64.4

**Total n**			**8341**	
**Weighted mean (%)**				**59.2**

Note. FIM, Functional Independent Measure.

**Table 2.** Stroke survivors with at least moderate dependency in activities of daily living (ADLs) at 1 year follow-up.

Study	Outcome Measure	Threshold	Sample Size (n)	Dependent Survivors (%)

Appelros (2007)	Barthel Index	<15/20	246	31.2
Broussy (2019)	Modified Ranking Scale	>2/6	161	29.6
De Campos (2017)	Modified Ranking Scale	>2/6	287	16.4
Jokinen (2015)	Modified Ranking Scale	>2/6	364	44.0
López-Cancio (2017)	Modified Ranking Scale	>2/6	143	53.8
Mar (2015)	Barthel Index	<90/100	250	40.4
Patel (2002)	Barthel Index	<15/20	619	36.2
Patel (2003)	Barthel Index	<15/20	136	36.0
Santus (1990)	Barthel Index	<75/100	76	46.1
Taub (1994)	Barthel Index	<15/20	225	11.0
Urbanek (2018)	Modified Ranking Scale	>2/6	1119	41.6
Verhoeven (2011)	Barthel Index	<18/20	92	38.0
Wafa (2020)	Barthel Index	<15/20	1961	24.1
Wolfe (2011)	Barthel Index	<15/20	1578	13.1
Wong (2014)	Modified Ranking Scale	>2/6	194	33.0

**Total** (n)			**7451**	
**Weighted mean** (%)				**32.9**

**Table 3.** Stroke survivors with severe or total dependency in activities of daily living (ADLs) at 1 year follow-up.

Study	Outcome Measure	Threshold	Sample Size (n)	Dependent Survivors (%)

Appelros (2007)	Barthel Index	<12/20	246	16.0
Broussy (2019)	Barthel Index	<12/20	161	12.7
Dhamoon (2009)	Barthel Index	<60/100	525	18.0
Gil-Salcedo (2022)	Modified Ranking Scale	>3/6	3718	27.3
Mar (2015)	Barthel Index	<60/100	250	20.4
Patel (2002)	Barthel Index	<10/20	619	9.4
Patel (2003)	Barthel Index	<10/20	136	15.4
Willey (2010)	Barthel Index	<60/100	246	15.9
Wong (2014)	Modified Ranking Scale	>3/6	194	19.6

**Total n**			**6095**	
**Weighted mean (%)**				**22.6**

**Table 4.** Stroke survivors who are moderately active in instrumental activities of daily living (IADLs) at 1 year follow-up.

Study	Outcome Measure	Threshold	Sample Size (n)	Dependent Survivors (%)

Appelros (2007)	Frenchay Activities Index	<30/45	246	78.8
Ayerbe (2011)	Frenchay Activities Index	<30/45	1403	79.7
Patel (2002)	Frenchay Activities Index	<30/45	619	85.7
Patel (2003)	Frenchay Activities Index	<30/45	136	88.2
Sveen (1996)	Frenchay Activities Index	<29/45	74	75.6

**Total n**			**2478**	
**Weighted mean (%)**				**81.5**

**Table 5.** Stroke survivors who are inactive in instrumental activities of daily living (IADLs) at 1 year follow-up.

Study	Outcome Measure	Threshold	Sample Size (n)	Dependent Survivors (%)

Appelros (2007)	Frenchay Activities Index	<15/45	246	46.3
Patel (2002)	Frenchay Activities Index	<15/45	619	40.4
Patel (2003)	Frenchay Activities Index	<15/45	136	72.7
van de Port (2006)	Frenchay Activities Index	<15/45	264	35.2
Wolfe (2011)	Frenchay Activities Index	<15/45	1578	38.8

**Total n**			**2843**	
**Weighted mean (%)**				**41.1**

**Table 6.** Baseline characteristics of the participants at their first interview for the Survey of Health, Ageing and Retirement in Europe (SHARE), when none of them had experienced a stroke, stratified by stroke-related status in the following waves.

Variables	Stroke Survivors (*N* = 2,143)	Stroke-Free Controls (*N* = 10,717)

**Age**, mean (SD)	66.9 (9.1)	66.9 (9.3)
**Sex**		
Female, n (%)	1052 (49.1)	5126 (47.8)
Male, n (%)	1091 (50.9)	5591 (52.2)
**Physical Activity**		
Hardly ever or never, n (%)	1193 (55.7)	6311 (58.9)
≥ Once a month, n (%)	950 (44.3)	4403 (41.1)
< Once a week, n (%)	1553 (72.5)	8014 (74.8)
≥ Once a week, n (%)	590 (27.5)	2703 (25.2)
**Functional Limitations**		
ADL, mean (SD)	0.2 (0.6)	0.2 (0.7)
IADL, mean (SD)	0.3 (0.8)	0.3 (0.9)
**Body Mass Index (kg/m^2^)**		
< 18.5 – Underweight, n (%)	155 (1.5)	590 (1.1)
18.5–24.9 – Normal, n (%)	3445 (33.9)	16275 (31.7)
25–29.9 – Overweight, n (%)	4176 (41.0)	22856 (44.5)
≥ 30 – Obese, n (%)	2401 (23.6)	11647 (22.7)
**Chronic Condition**		
< 2, n (%)	3423 (32.7)	22807 (43.5)
≥ 2, n (%)	7053 (67.3)	29676 (56.5)
**Education**		
Primary, n (%)	666 (31.1)	3027 (28.2)
Secondary, n (%)	1081 (50.4)	5415 (50.5)
Tertiary, n (%)	396 (18.5)	2275 (21.2)
**Country**		
Austria, n (%)	147 (6.9)	764 (7.1)
Belgium, n (%)	193 (9.0)	965 (9.0)
Czech Republic, n (%)	160 (7.5)	818 (7.6)
Denmark, n (%)	170 (7.9)	810 (7.6)
Estonia, n (%)	154 (7.2)	870 (8.1)
France, n (%)	161 (7.5)	815 (7.6)
Germany, n (%)	153 (7.1)	831 (7.8)
Greece, n (%)	106 (4.9)	506 (4.7)
Israel, n (%)	95 (4.4)	460 (4.3)
Italy, n (%)	161 (7.5)	768 (7.2)
Luxembourg, n (%)	20 (0.9)	94 (0.9)
Netherlands, n (%)	81 (3.8)	384 (3.6)
Poland, n (%)	73 (3.4)	368 (3.4)
Slovenia, n (%)	76 (3.5)	379 (3.5)
Spain, n (%)	141 (6.6)	673 (6.3)
Sweden, n (%)	167 (7.8)	812 (7.6)
Switzerland, n (%)	85 (4.0)	404 (3.8)

Note. ADL = activities of daily living, IADL = instrumental activities of daily living, SD = standard deviation.

**Table 7.** Results of the mixed-effects models testing the interaction between stroke-related status and physical activity (*once a week or less vs. more than once a week*) on limitations in activities of daily living (ADL) and instrumental activities of daily living (IADL).

	ADL		IADL	
	
Exposures	b (95 CI)	*p*	b (95 CI)	*p*

Intercept	−0.563 (−0.655 to −0.470)	< 2.0 × 10^−16^	−1.298 (−1.415 to −1.182)	< 2.0 × 10^−16^
Stroke	−0.111 (−0.144 to −0.078)	4.2 × 10^−11^	0.141 (0.100 to 0.182)	1.9 × 10^−11^
Physical Activity	0.351 (0.292 to 0.411)	< 2.0 × 10^−16^	0.410 (0.375 to 0.444)	< 2.0 × 10^−16^
Wave	0.018 (0.008 to 0.028)	3.5 × 10^−4^	0.018 (0.006 to 0.031)	0.004
Wave^2^	0.009 (0.008 to 0.010)	< 2.0 × 10^−16^	0.017 (0.151 to 0.189)	< 2.0 × 10^−16^
Age	0.008 (0.007 to 0.009)	< 2.0 × 10^−16^	0.014 (0.013 to 0.016)	< 2.0 × 10^−16^
Sex	0.072 (0.050 to 0.094)	1.5 × 10^−10^	0.203 (0.174 to 0.231)	< 2.0 × 10^−16^
Education				
Primary (vs. Secondary)	0.115 (0.090 to 0.140)	< 2.0 × 10^−16^	0.219 (0.187 to 0.251)	< 2.0 × 10^−16^
Tertiary (vs. Secondary)	−0.025 (−0.054 to 0.003)	0.081	−0.044 (−0.080 to −0.008)	1.7 × 10^−2^
Chronic Conditions	0.121 (0.106 to 0.136)	< 2.0 × 10^−16^	0.188 (0.170 to 0.206)	< 2.0 × 10^−16^
Stroke × Physical Activity	−0.083 (−0.149 to −0.018)	0.012	0.067 (−0.016 to 0.149)	0.116

Note. 95CI = 95% confidence interval, ADL = activities of daily living, IADL = instrumental activities of daily living.

**Table 8.** Results of the sensitivity analyses testing the interaction between stroke-related status and physical activity (*hardly ever or never vs. at least once a month*) on limitations in activities of daily living (ADL) and instrumental activities of daily living (IADL).

Exposures	ADL		IADL	
	
	b (95CI)	*p*	b (95CI)	*p*

Intercept	−0.494 (−0.589 to −0.399)	< 2.0 × 10^−16^	−1.042 (−1.168 to −0.917)	< 2.0 × 10^−16^
Stroke	−0.096 (−0.133 to −0.058)	6.2 × 10^−7^	−0.130 (−0.177 to −0.082)	< 2.0 × 10^−16^
Physical Activity	0.309 (0.256 to 0.362)	< 2.0 × 10^−16^	0.382 (0.314 to 0.449)	< 2.0 × 10^−16^
Wave	0.019 (0.009 to 0.029	1.4 × 10^−3^	0.021 (0.008 to 0.033)	0.001
Wave^2^	0.009 (0.007 to 0.010)	< 2.0 × 10^−16^	0.017 (0.015 to 0.019)	< 2.0 × 10^−16^
Age	0.007 (0.005 to 0.008)	< 2.0 × 10^−16^	0.012 (0.011 to 0.014)	< 2.0 × 10^−16^
Sex	0.062 (0.040 to 0.084)	4.2 × 10^−8^	0.192 (0.163 to 0.221)	< 2.0 × 10^−16^
Education				
Primary (vs. Secondary)	0.119 (0.104 to 0.133)	< 2.0 × 10^−16^	0.185 (0.167 to 0.203)	< 2.0 × 10^−16^
Tertiary (vs. Secondary)	0.116 (0.091 to 0.141)	< 2.0 × 10^−16^	0.223 (0.191 to 0.255)	< 2.0 × 10^−16^
Chronic Conditions	−0.027 (−0.056 to 0.002)	0.064	−0.049 (−0.085 to −0.012)	0.009
Stroke × Physical Activity	−0.086 (−0.144 to −0.028)	0.004	−0.065 (−0.138 to 0.009)	0.086

Note. 95CI = 95% confidence interval, ADL = activities of daily living, IADL = instrumental activities of daily living.

## Data Availability

The SHARE dataset is available at http://www.share-project.org/data-access.html and the DOIs for the waves used in the current study are: https://doi.org/10.6103/SHARE.w1.600, https://doi.org/10.6103/SHARE.w2.600, https://doi.org/10.6103/SHARE.w4.600, https://doi.org/10.6103/SHARE.w5.600, https://doi.org/10.6103/SHARE.w6.600, https://doi.org/10.6103/SHARE.w7.711, https://doi.org/10.6103/SHARE.w8cabeta.001.
